# Demographic disparities in animal law enforcement: rates and outcomes

**DOI:** 10.3389/fvets.2026.1885914

**Published:** 2026-08-12

**Authors:** Liana R. Moss, Kaleigh M. O'Reilly, Michelle Hansen, Amanda Arrington, Kevin N. Morris

**Affiliations:** 1 Graduate School of Social Work, Institute for Human-Animal Connection, University of Denver, Denver, CO, United States; 2 Humane World for Animals, Washington, DC, United States

**Keywords:** access to veterinary care, animal control, animal law enforcement, demographic disparities, policing inequity

## Abstract

Animal control is typically afforded the same enforcement powers and discretion as police in the United States. However, while there is a great deal of policing research, there is minimal research exploring demographic disparities in animal control enforcement. Aggregated animal control case and outcome data are well-positioned to examine demographic disparities in enforcement because they are linkable to census-level demographics due to the prevalence of cases at subjects' residences. The objectives of this study are to examine if the most marginalized communities experience higher numbers of animal control cases and if they are more likely to experience punitive enforcement. We analyzed a U.S. representative animal control dataset of 1,034,406 cases from 2015 to 2022 from five agencies. Geographic Information Systems (GIS) were used to link cases with their associated Social Vulnerability Index (SVI) scores, with higher scores identifying more marginalized communities. To control for inter-agency case volume variability, a weighted analysis of animal control outcomes was performed. An unweighted binomial logistic regression analysis controlling for multiple variables verifies the results of the weighted analysis. Results indicate that the most marginalized communities experience the preponderance of cases and higher rates of punitive outcomes. Racial & Ethnic Minority Status and Socioeconomic Status SVI domains primarily influence the disparities, even when controlling for case type. Combined results highlight racial and socioeconomic inequities in animal law enforcement that hold wider implications for the intersections between animal control and access to veterinary care.

## 1 Introduction

Higher rates of crime and policing enforcement in historically and systemically marginalized communities (typically identified by high percentage of racial and ethnic minority individuals and/or low socioeconomic status; hereafter referred to as “marginalized communities”) in the U.S. are well established (1–6). Unfortunately, while commonly afforded the same discretionary powers as police, there is minimal research exploring if these same phenomena exist within the animal control field (7, 8). In context, it is estimated that 20 million pets live with people whose income level is below the U.S. poverty line, 70% of pets living in poverty have never seen a veterinarian, and 28% of all pet homes report an inability to obtain veterinary care and even access to basic pet-keeping resources (9–15). In addition to public safety/health (e.g. dangerous dogs, rabies control) and code compliance (e.g., odor, animal waste, nuisance animals), the focus of many animal laws and ordinances on animal welfare/health (e.g., cruelty/neglect cases) combined with a systemic lack of access to veterinary care in marginalized communities, complicates understanding how animal law enforcement engages with the communities they serve (16, 17).

Animal control officer responsibilities differ somewhat between jurisdictions, but typically, officers are tasked with enforcing local and state laws related to the control and welfare of domestic animals and, sometimes, livestock and wildlife (16). Animal control services are delivered through a variety of organizational arrangements with local governments which are responsible for deciding if the program will be administered by the county or municipality itself, or outsourced through a contract to another organization, such as a private animal shelter (16). As a component of policing, animal control officers employed by these agencies are trained in law enforcement, some court procedures, and can carry weapons (e.g., firearms, tasers, pepper spray) dependent on the jurisdiction (7, 16). They have discretion to enforce local animal laws through non-punitive approaches (e.g., warning, return to owner, provide resources) or punitive measures (e.g., citations, fees, fines, and search and seizure of property [i.e., owned animals]) (7, 16).

Previous peer-reviewed research on animal control in the U.S. has largely focused on animal welfare issues, such as instances of animal cruelty and neglect and their correlation with other types of violent crime, rates of animal shelter intakes and outcomes, factors influencing the surrender of animals at shelters, and perspectives of animal control officers on enforcement practices (7, 18–25). Only a few studies have reported jurisdiction-specific and crime-specific data illustrating that more animal-related crime and penalties occur in marginalized communities (19, 25, 26). While research exists on enforcement patterns for other sectors of the policing system on at least subsets of the case types (27, 28), no comprehensive studies have been published that establish patterns of animal control enforcement across the complete set of case types or the demographics of an entire jurisdiction or multiple jurisdictions.

For this research article, we constructed a dataset of over one million animal control cases with U.S. national census representation from five large, anonymous animal control agencies. This unprecedented dataset includes every case from 2015 to 2022, with details on case type, case outcome type, and location. Geographic Information Systems (GIS) were used to link each case with its associated Social Vulnerability Index (SVI) score as a measure of community marginalization. We then conducted a variety of analyses to answer two primary research questions (RQs). RQ1: Compared to the least marginalized communities, are the most marginalized communities experiencing higher numbers of animal control cases? RQ2: Compared to the least marginalized communities, are the most marginalized communities more likely to experience punitive enforcement from animal control?

## 2 Materials and methods

### 2.1 Design

This study utilized a retrospective longitudinal quantitative design to examine the patterns of animal control enforcement and their relationships to community demographics from 2015 through 2022. Data covering this period enabled an assessment of general patterns in the field, including during the COVID-19 pandemic that impacted community enforcement practices starting in 2020. Given the inter-agency case volume variability, a weighted analysis was employed to adjust for the overrepresentation of certain agencies in the sample and ensure a greater generalizability of the results to the U.S. animal control agency population. An unweighted analysis of case outcomes was also utilized to control multiple variables and verify the results of the weighted analyses.

### 2.2 Data collection

#### 2.2.1 Animal control data

A nationally representative U.S. sample of animal control agencies with detailed case data, including location, from 2015 through 2022, was collected for this study. Animal control agency recruitment and all activities for the study were carried out under an Institutional Review Board-approved protocol at the University of Denver. Selection criteria included urban, municipal animal control agencies or non-profit agencies with a municipal government contract from different geographic locations within the U.S. Five animal control agencies, referred to as agencies A-E, located in all four U.S. Census Regions (West, Midwest, South, and Northeast) were identified as fitting these criteria and willing to provide their case data (29). During the study period, the agencies served an average population of nine million people and physical area of 10,000 mi^2^ within 2,094 census tracts each year (16,748 census tracts in total).

Given the potentially sensitive nature of the study and inclusion of address data that identify the location where cases occurred, the Data Use Agreements (DUAs) executed between the university and the animal control agencies were designed to ensure anonymity by including that all data would be deidentified and only analyzed in aggregate. One agency requested the execution of a City Public Records Request in lieu of a DUA in order to streamline the data transfer process. Participating agencies were asked to deidentify all case data that they provided, except for information regarding case location (street address including zip code). Animal control agencies transferred their case data using secure, individualized links to the password-protected, HIPAA and FERPA compliant database, REDCap, hosted at the University of Denver (30, 31). Any additional identifiable data (beyond location information) that were inadvertently shared by participating agencies were immediately deleted by research personnel. All data were then downloaded and stored in a password-secured Microsoft OneDrive folder (Online Version).

The data include every animal control case from 2015 to 2022 with details on the date, location, case type, case outcome type, and notes from the case. The aggregate raw data consisted of 1,203,676 cases. Those involving wildlife (e.g., opossums, raccoons, squirrels, coyotes, wild birds), livestock (e.g., pigs, cows, goats, horses), and small domestic animals (e.g., ferrets, hamsters, domestic rabbits) were removed, unless they also included at least one dog or cat, as these types of cases are not responded to by all the agencies in the study. Of the remaining 1,076,280 cases in the dataset, another 41,874 were removed during GIS mapping because they didn't include a complete address or were located outside of the agency's jurisdiction. The final dataset of 1,034,406 cases focused on dogs and/or cats was then sorted into 17 case types and 20 outcome types developed with expert input (e.g., animal control agency leadership and officers, Humane World for Animals' Pets for Life) as the minimum number of categories to encompass all cases and outcomes across agencies (Supplementary Table S1). The outcomes were further characterized as Punitive, Non-Punitive, or Neither based on legal definitions of outcomes.

#### 2.2.2 Social vulnerability index (SVI)

Animal control agencies typically do not track the demographics of individuals with whom they interact. Therefore, the Centers for Disease Control and Prevention (CDC) and the Agency for Toxic Substances and Disease Registry (ATSDR)'s SVI was used to quantify the demographics and social environments of communities where animal control cases occurred. Social vulnerability is understood as the socioeconomic and demographic elements affecting the resilience of communities (32). Though originally created for use in disaster planning, multiple studies validate the use of SVI to also characterize the marginalization of a community (32–35). Total SVI ranks the marginalization of each census tract on a 0 to 1 scale, with 0 being the least and 1 being the most marginalized areas and is calculated down to three decimal places. It is comprised of 16 census variables organized in four domains: Socioeconomic Status (SES), Racial & Ethnic Minority Status (RE), Household Characteristics (HC), and Housing Type & Transportation (HTT). Before 2020, the RE domain was known as Minority Status & Language. To maintain analytical consistency, only the “Minority” variable within the RE domain was utilized in analyses for all years because “Language” was not included in that domain throughout the entire study period.

### 2.3 Data analysis

#### 2.3.1 ArcGIS

The location of each animal control case was geocoded onto shapefiles of the five agencies' jurisdiction using ArcGIS software (36). Shapefiles were adjusted annually as needed to account for any changes in jurisdiction based on public municipal contract records. Cases were removed from analysis if they were unable to be accurately geocoded due to having incomplete addresses or a location outside the scope of the agency's jurisdiction (*n* = 41,874). ESRI Geodatabase files containing SVI data at the census tract level were downloaded directly from the CDC/ATSDR SVI website for each biennial period starting in 2014 and for each U.S. State in which an animal control agency was located. Shapefiles with geocoded animal control cases were then overlaid with the SVI data, producing data files with each animal control case type and outcome linked to its associated SVI score. All data analyses were conducted using this dataset. Maps produced using GIS will not be publicly shared to uphold the anonymity of participating animal control agencies and to protect the privacy of individuals' addresses associated with cases in the sample.

#### 2.3.2 SPSS

Statistical analyses were performed using IBM SPSS Statistics 28.0.1.1 (37).

#### 2.3.3 Case volume analysis

**Figure 1:**
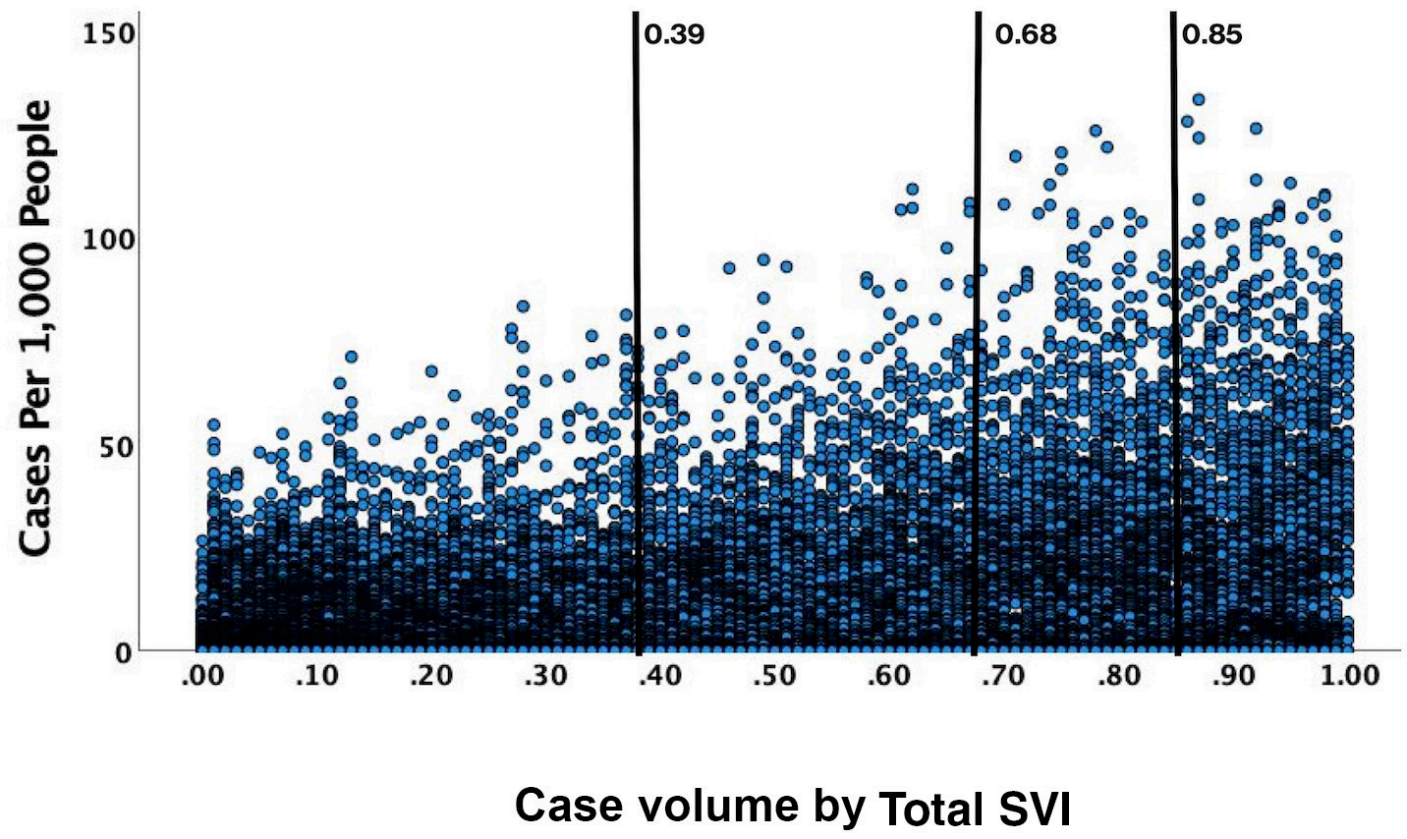
The distribution of animal control cases per 1,000 people per census tract (*n* = 16,748) by Total SVI. 137 extreme outliers removed via IQR analysis. Lines at 0.39 SVI, 0.68 SVI, and 0.85 SVI indicate where Q1, Q2, and Q3 occur, respectively.

To assess if the most marginalized communities are experiencing higher case volume from animal control than the least marginalized communities (i.e., RQ1), we examined the number of cases per 1,000 people per census tract (*n* = 16,748) and the SVI scores for each census tract (Figure 1). Outliers were assessed through an Interquartile Range (IQR) analysis, which defines outliers as values at least 1.5 × the IQR above the third quartile or below the first quartile. This IQR analysis can be utilized even when data are not normally distributed (38). Case volume distribution by Total SVI and each of its domains was examined in two ways: (1) as the SVI score at which Q1 = 25%, Q2 = 50%, and Q3 = 75% of the total case volume occur; and (2) as a percentage of the total number of cases that occur within each SVI quartile. While there is no standard way to analyze SVI, we chose to mirror previously published studies by dividing the SVI census tracts into quartiles for analysis [0–0.25 (Low), 0.26–0.50 (Moderate), 0.51–0.75 (High), 0.76–1 (Very High)] (39–41).

#### 2.3.4 Weighted analysis of outcomes

To assess if the most marginalized communities are more likely to experience punitive enforcement from animal control than the least marginalized communities (i.e., RQ2), we examined the number of Punitive and Non-Punitive outcomes per 1,000 people and the SVI of each census tract. A unique characteristic of this dataset is the case outcome detail that provides information on animal control interactions beyond typically tracked punitive outcomes, such as citations or seizures/impoundments of an animal. Our data include Punitive, Non-Punitive, and Neither outcomes that add detail to the overall nature of enforcement practices occurring in communities. However, given prior research in human policing that indicates there are more cases/interactions with human police in marginalized communities, researchers hypothesize that this same pattern will emerge in animal law enforcement data. Therefore, a relationship must be established between Punitive and Non-Punitive outcomes across communities in order to establish the nature of enforcement. We calculated the number of Punitive outcomes per 1,000 people in each census tract in the sample and divided this by the number of Non-Punitive outcomes per 1,000 people. This creates the Punitive/Non-Punitive Outcome Ratio (PNPOR) for each census tract. Given the inter-agency case volume variability, a weighted analysis was employed to adjust for the overrepresentation of certain agencies in the sample and ensure a greater generalizability of the results to the U.S. animal control agency population. We aggregated census tract PNPOR ratios onto an SVI 100-point scale for each organization, effectively weighting the PNPOR equally for all five organizations. Outliers were again assessed through an Interquartile Range (IQR) analysis (38). We again chose to mirror many current studies and divide SVI census tracts into quartiles for analysis (39–41). In addition, ANOVA difference between means analyses with a Bonferroni correction were used to evaluate the statistical significance of differences between means across the SVI quartiles.

#### 2.3.5 Unweighted analysis of outcomes

As a verification of the weighted analyses, a binomial logistic regression model was employed as an unweighted analysis of animal control case outcomes as it allows for the controlling of multiple variables and has been utilized in prior literature to analyze outcomes across SVI (42, 43). A logistic regression model predicts the odds of a binary/dichotomous dependent/response variable (outcome) based on multiple independent predictor/explanatory variables without an assumption of normality to conduct a binomial logistic regression analysis (44). In the context of this study, the response variable is the existence of a Punitive outcome, and the predictor variables, or covariates, are the agency, year, case type, and SVI. Year data were included in the model as a categorical variable because it is not appropriate to assume a continuous relationship, and this allows for the control of year-specific effects. The presence of these predictor variables allows the researchers to establish their effect on the odds of a Punitive outcome and to assess the effect of SVI on the odds of a Punitive outcome when all predictor variables are held constant within the model. The reference categories chosen for the binomial logistic regression models were Bite (case type), 2015 (year), and Agency B (agency) because the median ratio of Punitive to Non-Punitive outcomes across case types in our sample is the Bite case type (Supplementary Table S2), the start of our study period is 2015, and the percentage of cases Agency B contributed (22.14%; Supplementary Table S2) being roughly representative of the mean percentage of cases contributed by agencies. As with the weighted analysis, Neither outcomes were omitted for this analysis.

## 3 Results

This study's sample consists of 1,034,406 animal control cases across five agencies serving primarily urban jurisdictions over 8 years (Supplementary Table S3). During the study period, the agencies served an average population of nine million people and physical area of 10,000 mi^2^ within 2,094 census tracts each year (16,748 census tracts in total). Inter-agency case volume varied with the total number of animal control cases in the study period ranging from min = 25,335 or 2.45% to max = 547,188 or 52.90% of cases. In the aggregate sample, there was a mean (*M*) of 142,645 cases per year (SD = 8,981) from 2015 to 2019. In 2020, case volume decreased 31.3% from the prior 4-year mean to 98,027 cases. This significant case volume decrease is likely due to the onset of the COVID-19 pandemic, which deeply impacted the functioning of U.S. municipal operations, including animal control (13, 45–48). Due to this significant difference in case volume in 2020, the mean number of cases per year for the total study period, 2015 to 2022, is 129,301 (SD = 20,069).

Of the 1,034,406 animal control cases, 365,754 or 35.36% resulted in a Punitive outcome, 410,693 cases or 39.70% resulted in a Non-Punitive outcome, and 257,959 cases or 24.94% resulted in a Neither outcome. The latter outcome type was largely driven by the “Unable to make contact/locate” category, which largely represents unsuccessful responses to reports of stray animals. An average Total SVI score of 0.50 and a median Total SVI score of 0.51 indicate that the 16,748 census tracts in the dataset are equally distributed across the scale (Supplementary Table S4).

### 3.1 RQ1: case volume by SVI

A quartile analysis, which examined the number of cases per 1,000 people per census tract (*n* = 16,748) and its associated SVI score, was used to assess if the most marginalized communities are experiencing higher case volume from animal control than the least marginalized communities (Figure 1). For case volume distribution by Total SVI, Q1, Q2, and Q3 occur at Total SVI scores of 0.39, 0.68, and 0.85, respectively. Via interquartile range (IQR) analysis, 137 extreme outliers (< 0.82% of census tracts in the sample) had been removed for this analysis. Based on the approximately normal distribution of SVI data, established by the dataset's Total SVI mean and median (Supplementary Table S4), the results indicate a skew in case volume in which census tracts with the highest Total SVI scores experience relatively more cases than census tracts with the lowest Total SVI scores. The same analysis for all four SVI domains [Racial & Ethnic Minority Status (RE), Socioeconomic Status (SES), Household Characteristics (HC), and Housing Type & Transportation (HTT)] identified the RE domain as the strongest contributor to the skew in case numbers measured in Total SVI (Table 1).

**Table 1:** Summary of case volume distribution by Social Vulnerability Index (SVI).

SVI domain	# of outliers	Animal control cases per 1,000 people by SVI quartile								SVI score at which 25%, 50%, & 75% of animal control cases occur		
		Low		Moderate		High		Very High				
		#	% of total	#	% of total	#	% of total	#	% of total	*Q*_1_	*Q*_2_	*Q*_3_
Total SVI	137	41,996.14	16.55%	43,206.37	17.02%	68,227.37	26.88%	100,394.24	39.55%	0.39	0.68	0.85
RE	223	19,055.94	7.66%	23,641.53	9.50%	40,641.51	16.33%	165,482.27	66.51%	0.65	0.87	0.95
SES	109	40,754.65	15.90%	45,233.91	17.64%	62,303.47	24.30%	108,064.78	42.15%	0.40	0.68	0.88
HC	187	54,003.91	21.60%	53,381.83	21.35%	65,494.47	26.19%	77,165.47	30.86%	0.30	0.57	0.80
HTT	187	59,569.77	23.74%	60,989.95	24.31%	71,386.36	28.46%	58,928.20	23.49%	0.26	0.52	0.74

The distribution of animal control cases for Total Social Vulnerability Index (SVI), as illustrated in Figure 1, and each SVI domain (RE, SES, HC, HTT). The four SVI domains are Racial & Ethnic Minority Status (RE), Socioeconomic Status (SES), Household Characteristics (HC), and Housing Type & Transportation (HTT). For each analysis, the summary details: (1) the total number of outliers removed, (2) the number of cases and the percentage of the total represented in each quartile of SVI [0–0.25 (low), 0.26–0.50 (moderate), 0.51–0.75 (high), 0.76–1 (very high)], and, (3) the SVI score at which Q1, Q2, and Q3 occur.

The results of these initial quartile analyses are reinforced by an analysis of the percentage of the total number of cases that occur within each SVI quartile for each SVI domain. The same outliers removed via IQR analyses for the previous analysis were excluded again. Notably, the Very High SVI quartile accounts for 39.55% of cases by Total SVI, 42.15% of cases by SES, and 66.51% of cases by RE, whereas the HC and HTT domains only accounted for 30.86% and 23.49%, respectively (Table 1). The number of outliers removed was < 1.33% of cases in all analyses.

### 3.2 RQ2: case outcomes by SVI

#### 3.2.1 Weighted analysis

To assess if the most marginalized communities are more likely to experience punitive enforcement from animal control than the least marginalized communities, we examined the outcomes using a Punitive to Non-Punitive ratio and the SVI score of each census tract (Table 2). We calculated the number of Punitive outcomes per 1,000 people in each census tract in the sample and divided this by the number of Non-Punitive outcomes per 1,000 people in that census tract to create the Punitive/Non-Punitive Outcome Ratio (PNPOR). We then aggregated census tract PNPOR ratios onto the SVI scale for each organization, effectively weighting the PNPOR equally for all five organizations. Via IQR analysis, two extreme outliers (< 0.40% of data) were identified and removed for the analysis of PNPOR by Total SVI. To compare the nature of outcomes across SVI, the average PNPOR for each Total SVI quartile was calculated as 0.64, 0.69, 0.82, and 0.86 for the Low, Moderate, High, and Very High SVI quartiles, respectively. There was a statistically significant difference between SVI quartiles as determined by one way ANOVA [*F*_(3, 499)_ = 8.879, *p* < 0.001]. A Bonferroni correction revealed that the PNPORs for the High quartile (0.82 ± 0.451, *p* = 0.002) and the Very High quartile (0.86 ± 0.467, *p* < 0.001) were statistically significantly larger compared to the Low quartile (reference) PNPOR (0.64 ± 0.311). There was no statistically significant difference between the Low and Moderate quartiles (*p* = 1.00; Supplementary Table S5). These results indicate a skew in case outcomes in which the Very High Total SVI quartile experiences an average PNPOR that is 1.35 times greater than Low Total SVI quartile. This same analysis was repeated for all four SVI domains (Table 2; Supplementary Tables S6–S9). In all cases, outliers account for < 0.40% of data, and undefined data (PNPOR with a 0 in the denominator) account for < 0.40% of data. These analyses identified the RE domain as the strongest contributor of the skew in case outcomes measured in Total SVI.

**Table 2:** Summary of results of weighted analysis of case outcomes by Social Vulnerability Index (SVI).

	SVI domain				
SVI quartile	Total SVI	RE	SES	HC	HTT
Low	0.64	0.61	0.63	0.69	0.74
Moderate	0.69	0.63	0.71	0.78	0.79
High	0.82^*^	0.77^*^	0.82^*^	0.84^*^	0.80
Very High	0.86^*^	0.85^*^	0.86^*^	0.84^*^	0.85
Difference between Very High and Low quartiles	1.35	1.40	1.35	1.22	1.15

The average PNPOR for each SVI quartile for Total SVI and all four SVI quartiles is shown, with higher PNPORs indicating more punitive enforcement. The four SVI domains are Racial and Ethnic Minority Status (RE), Socioeconomic Status (SES), Household Characteristics (HC), and Housing Type and Transportation (HTT). SVI quartiles are defined as 0–0.25 (Low), 0.26–0.50 (Moderate), 0.51–0.75 (High), 0.76–1 (Very High).

(^*^) indicates the result is a statistically significant different mean from the Low quartile (*p* < 0.05). The magnitudes of the differences between the Very High and Low quartiles for Total SVI and four SVI domains are shown.

#### 3.2.2 Unweighted analysis

A binomial logistic regression analysis, with Punitive and Non-Punitive outcomes serving as the binary outcomes, was also employed to determine the odds and probability of a Punitive outcome across SVI. Total SVI, SES, RE, HC, and HTT were analyzed separately in five separate models as each of these variables is significantly correlated (*p* < 0.001) with one another in a Pearson Correlation analysis (Supplementary Table S10). The reference categories chosen for the binomial logistic regression models were Bite (case type), 2015 (year), and Agency B (agency). Importantly, this allows for the control of the effects of case type, year, and agency with the model.

**Figure 2:**
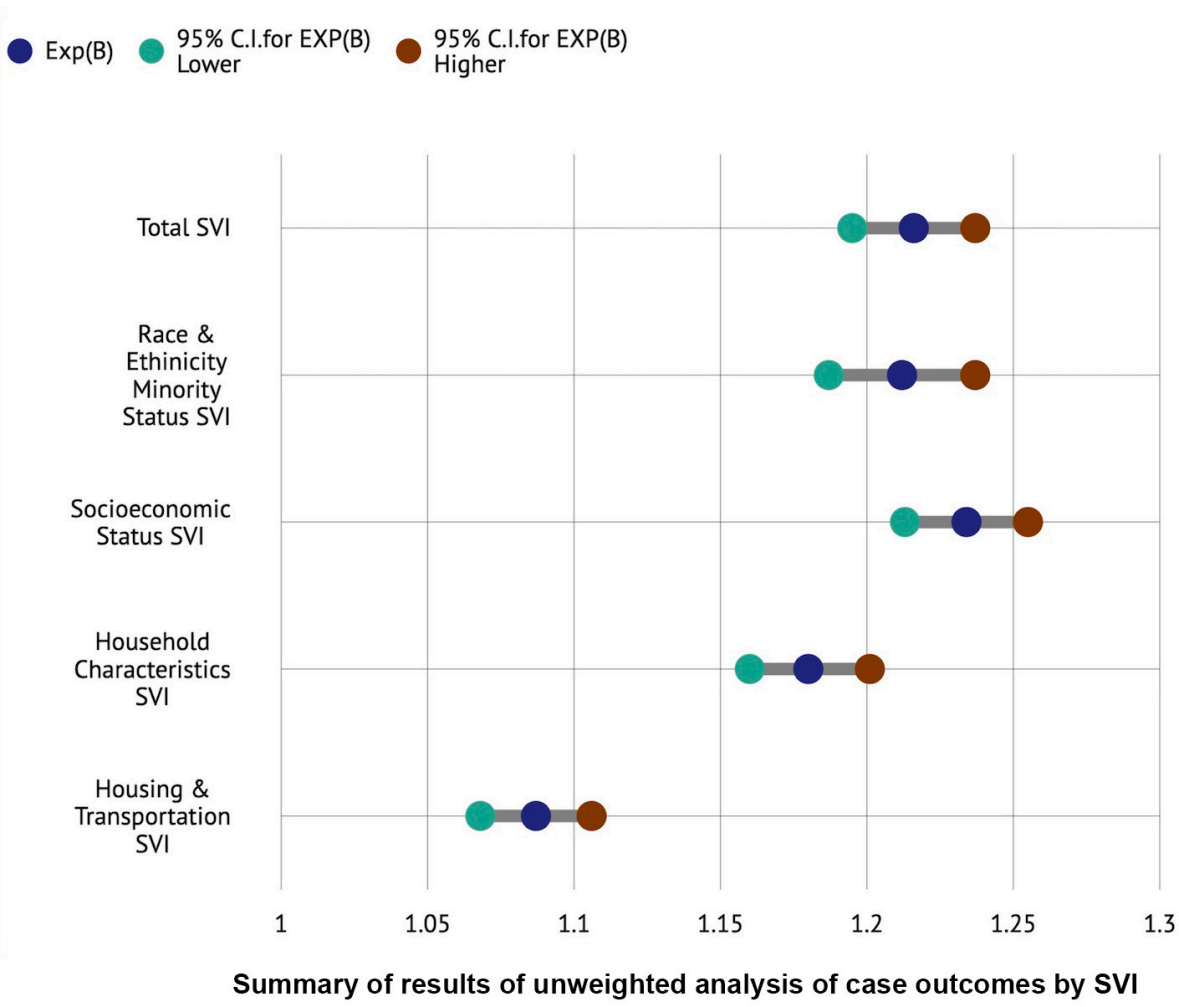
The relationship between SVI domains and Punitive outcomes is measured by the odds ratio.

The odds of a Punitive outcome increase as Total SVI increases [Supplementary Table S11; *p* < 0.001, OR = 1.22, 95%CI (1.20, 1.24)]. This same analysis was repeated for each individual SVI domain. These analyses revealed that the likelihood of receiving a Punitive outcome increases when each domain increases, with RE [*p* < 0.001, OR = 1.21, 95% CI (1.19, 1.24)], SES [*p* < 0.001, OR = 1.23, 95%CI (1.21, 1.26)], HC [*p* < 0.001, OR = 1.18, 95% CI (1.16, 1.12)], and HTT [*p* < 0.001, OR = 1.09, 95% CI (1.07, 1.11); Figure 2; Supplementary Tables S12–S15]. Finally, a model including the interaction of Total SVI and agency was used to assess the uniformity of these findings across geographic regions and found that the effect associated with Total SVI varied somewhat by agency, but the direction of the effect is consistent (Supplementary Table S16).

## 4 Discussion

Our analysis of over one million animal control agency cases with U.S. national geographic representation across the entire array of case types and outcomes provides unique insight into how this sector of the broader policing system interacts with the jurisdictions they serve. Our finding that the most marginalized communities experience more enforcement is consistent with previous policing research (1–6). This higher animal control enforcement rate is strongly influenced by the RE and SES domains of SVI, with half of the cases occurring in the 13% and 32% most racially/ethnically and socioeconomically marginalized census tracts, respectively, in our quartile analysis of case volume. While race/ethnicity and socioeconomic status have been linked by a substantial body of research (49–54), the effect of the RE domain is prominent in our analysis, with a quarter of cases occurring within the top 5% of the RE domain. Our research further shows that the most marginalized communities not only experience more enforcement but also higher rates of punitive outcomes. Again, the RE and SES domains most influence the overall skew in our weighted analysis and are most highly correlated with an increased likelihood of a punitive outcome in our unweighted analysis. The consistency of results generated by our two analytical approaches provides higher confidence that these correlations between outcomes and Total SVI and its RE and SES domains are accurate, and the large number of cases, broad geographic distribution, and consistency of results across all five agencies support some generalizability of the findings, at least within urban jurisdictions.

While higher rates of crime and policing enforcement in historically and systemically marginalized communities in the U.S. are well established, conclusive evidence supporting an explanation for the primary cause of this phenomenon remains a subject of intense research and debate (55–57). This largely reflects the difficulty in obtaining and aggregating police data due to: (1) the large variety of criminal and civil infractions found in state or local laws under police jurisdiction (i.e., crimes against people, crimes against property, crimes against society) (58); (2) the inconsistencies in data collection methods between different jurisdictions (with local and nationally mandated reporting limited to a few subject areas) (27, 28, 59–61); and, (3) a reluctance of U.S. police agencies to collect and share comprehensive datasets (27, 60, 62). Though many of these same issues are present in the animal control sector of the policing system, animal control is better positioned to evaluate demographic disparities in enforcement in a more generalizable manner for several reasons. First, animal control covers a smaller subset of civil and criminal laws, thereby facilitating the standardization of aggregate data. Second, animal control officers are typically afforded the same discretionary powers as police, making it possible to assess enforcement biases (7, 8). Third, policing research has typically focused on where an enforcement action occurs. While many police enforcement actions (e.g., traffic violations, theft) often occur in different census tracts from where subjects of cases reside, animal control officers most often respond to a subject's home address (e.g., animal neglect, animal nuisance) (16, 27). Even when it comes to Stray/At Large cases, 70% of stray dogs are found within one mile from their owner's home address, and 42% are found within 400 feet, a distance equivalent to one city block (63). Therefore, a dataset of multiple animal control agencies' activities is more aggregable and linkable to relevant enforcement patterns tied to census tract-level demographics than a dataset of police cases.

One potential rationale to explain higher numbers of cases in certain communities is that animal control agencies, like the police, often reactively respond to cases where they are dispatched and do not choose where they respond in the jurisdiction they serve (64). However, our findings show that the inequitable nature of enforcement extends beyond the frequency of cases to the actual application of different outcomes in different parts of a jurisdiction. This inequity in outcomes persists even when controlling for case type in the binomial logistic regression model, which suggests that the explanation that there are more “serious” case types occurring in more marginalized communities, thus deserving more punitive outcomes, is not a viable explanation for the inequitable pattern of enforcement. While we conducted the analyses with the aggregate data to ensure anonymity of the participating agencies, it's important to note that different agencies have different patterns of enforcement, with some being more/less punitive and some being more/less equitable. However, even agencies that issue more non-punitive outcomes than punitive outcomes on average inequitably apply punitive outcomes to their most marginalized community members (Supplementary Table S16).

Our study also identified the SES SVI domain as a strong driver of the results across our analyses of enforcement rates and outcomes, which is strongly linked to race/ethnicity and police overenforcement by previous research (49–54). Recent research estimates that 129 million Americans live in areas considered to be a veterinary desert (65), 52% of pet owners have not visited a veterinarian within the past year, even when they felt their pet needed care, or have declined care that was recommended by a veterinarian, and 71% of these pet owners say it is because they could not afford care or that it was not worth the cost (66). Unfortunately, veterinary care is becoming more expensive, with the cumulative U.S. veterinary care inflation (44%) far exceeding the cumulative U.S. inflation (26%) in 2025 and the average annual cost of care reaching as high as $3,000 per pet (67). With 20 million pets living with people whose income level is below the U.S. poverty line, this systemic lack of access to animal care resources in low socioeconomic level communities can create barriers for individuals to comply with animal control laws (10). Moreover, the inequitable application of animal control enforcement in low socioeconomic level communities can instigate and perpetuate negative health outcomes for the very animals the system is tasked with protecting (68). Inequitably confiscating and impounding animals in animal shelters, rather than instituting problem-solving strategies or addressing root-cause issues with access to veterinary care, reduces shelter capacity to care for animals in crisis, contributes to higher euthanasia rates, lower return to owner rates, and extends animal lengths of stay (68–70). To address these systemic inequities in access to services, some animal control agencies have transitioned from a solely enforcement-based approach to more supportive models of animal control (7, 68, 71–73). However, given our findings, even animal control enforcement agencies employing these strategies must examine and ensure that these models are applied equitably across all communities in the jurisdiction they serve.

Many of the punitive outcomes included in this study result in the imposition of fines, fees, civil penalties, and surcharges, though the exact amounts vary by jurisdiction and case types. Across the U.S., the use of legal financial obligations (LFOs) has risen, often as a method of increasing municipal revenues rather than as a tool for public safety (74, 75), and disproportionately impact communities that are the most racially and socioeconomically marginalized (74–79). In already marginalized communities, LFOs create difficult-to-escape cycles that hinder economic mobility and perpetuate poverty. On an individual level, nonpayment of an LFO can lead to driver's license suspension, restriction of voting rights, harm to credit, incarceration, and the extension of probation or parole. The cascading effect of LFOs can also impact employment, food security, and the ability to obtain housing. On a broader level, the inequitable distribution of LFOs damages judicial credibility, builds distrust of law enforcement officers within communities, and can create unethical conflicts of interest (74–78, 80). Even so, there are many organizations and individuals within the animal protection movement that advocate for stricter laws and harsher punishment related to animal crimes, including pursuing mandatory minimum sentences, the prosecution of juveniles as adults, more felony prosecutions, and offender registries (81). Given the inequitable application of punitive outcomes identified in this study and the serious consequences of inequitable enforcement, caution should be exercised against broadscale criminal penalties within animal laws. Our findings highlight a critical need to develop evidence-based policies and practices that balance proactive prevention of harm to animals, while addressing the downstream harms against individuals, families, communities, and the human-animal bond caused by overenforcement and inequitable enforcement.

There are no simple nor solitary solutions to the complex, multilevel problem of inequitable enforcement. However, there are policy and practice solutions proposed in the scientific literature and/or employed effectively in policing to reduce demographic bias in law enforcement. Such interventions include robust, thoughtful community engagement practices, reduction of discretionary officer actions (e.g., indiscriminate patrols, “aggressive preventative patrols,” door-to-door compliance checks), and intentional avoidance of revenue-generating policing (7, 75, 82–86). In addition, animal law enforcement data collection, specifically including nuanced demographic data of people who intersect with animal control, must be standardized. These data should also be rigorously analyzed, transparently available to the public, and externally overseen to attempt to avoid endogenous system bias, so evidence-based policies and practices that create more equitable outcomes for both people and animals can be identified and implemented (7, 27, 87). Moreover, policing and animal control research suggests that moving beyond surveillance and traditional enforcement actions toward problem-solving strategies may be more effective, equitable, and ethical (7, 82, 88, 89). Ultimately, as in human policing, the most meaningful animal control reforms “must be tailored to and reflect the realities of local conditions [...] concrete recommendations should come not from a single expert, but from a collaborative team of experts comprising social scientists, law enforcement, public officials, residents, and other stakeholders” (85).

### 4.1 Limitations

While this study contributes substantially to our understanding of disparities in the policing system based on animal control enforcement data and jurisdictional demographics, it has several limitations. First, while the inclusion of five agencies from all four census regions provides some generalizability of the findings, the participating agencies served predominantly large, urban jurisdictions, so it remains unclear if the results found here are generalizable to enforcement practices in rural areas. Second, it is important to note that while current research indicates SVI remains a valid metric of vulnerability, the CDC and ATSDR occasionally make slight changes and updates to the methodology used to generate the census tract-level scores (90, 91). We accounted for these changes by consistently using the 2022 SVI definitions across the longitudinal dataset. Third, the data were collected by each animal control agency to track enforcement activities according to agency-specific priorities. This was reflected in differing data collection categories, which had to be standardized by the research team, which might account for some of the interagency variation in case volume and punitive outcome skews. Further, some decisions to include an outcome in one category or another could be argued either way. For example, “Surrender” was included in the Non-Punitive outcomes category for analysis to align with the definition of surrender as the voluntary signing-over of ownership and responsibility to the animal control agency, as stated in the laws of the study agency jurisdictions. However, the researchers acknowledge that animal control citations can potentially be used as a tool of threat or coercion to surrender an animal (i.e., the owner surrenders the animal to avoid citations and/or further legal action), so these situations may not always be entirely voluntary in nature (71, 92). Finally, the willingness of the five participating agencies to provide data, which indicates an interest in ascertaining how they are interacting with the jurisdictions they serve, likely introduces a bias in the dataset. For each participating agency, approximately two others declined to provide their data. Therefore, this study's findings might be less skewed toward higher case numbers, punitive outcomes, and inequitable enforcement than a randomly selected group of agencies for which voluntary data acquisition would not be required.

## Data Availability

The availability of the raw data from this research is restricted via Data Transfer and Use Agreements (DUAs), which were executed between the University of Denver and the anonymous animal control agencies that agreed to participate in this study. In order to make the data available while maintaining the anonymity of the participating agencies, a copy of our aggregate, deidentified, and rounded (to the second decimal place) data will be provided upon request. Requests to access these datasets should be directed to Kevin N. Morris, kevin.morris@du.edu.
