# Supplementary material for: Demographic disparities in animal law enforcement: rates and outcomes
Source: Front Vet Sci. 2026 Aug 12;13:1885914. doi: 10.3389/fvets.2026.1885914 (PMC13506396; doi:10.3389/fvets.2026.1885914)
Supplement: Supplementary file 1 [file Table_1.docx]

Supplementary Material

# 1 Supplementary Tables

**Table S1.** Animal control data categories.

Table S2. Descriptive statistics of outcomes by case type.

*Note.* The Bite case type (A) is associated with the Median ratio (C) and represents the third greatest number of outcomes (B) across case types.

**Table S3.** Descriptive statistics of the animal control case dataset.

Table S4. Descriptive statistics of sample SVI data.

**Table S5.** Difference between mean PNPOR of Total SVI Quartiles.

*Note.* For between-group post hoc test, significant difference between quartiles is denoted by **p* <0.05.

**Table S6.** Difference between mean PNPOR of RE SVI Quartiles.

*Note.* For between-group post hoc test, significant difference between quartiles is denoted by **p* <0.05.

**Table S7.** Difference between mean PNPOR of SES SVI Quartiles.

*Note.* For between-group post hoc test, significant difference between quartiles is denoted by **p* <0.05.

**Table S8.** Difference between mean PNPOR of HC SVI Quartiles.

*Note.* For between-group post hoc test, significant difference between quartiles is denoted by * *p* <0.05.

**Table S9.** Difference between mean PNPOR of HTT SVI Quartiles.

*Note.* For between-group post hoc test, significant difference between quartiles is denoted by **p*<0.05.

**Table S10.** Pearson Correlation Analysis of SVI Total and Domains.

*Note.* Significant relationships are denoted by **p<0.01.

**Table S11.** Binomial logistic regression analysis of the odds of a punitive outcome by Total SVI.

**Table S12.** Binomial logistic regression analysis of the odds of a punitive outcome by RE.

Table S13. Binomial logistic regression analysis of the odds of a punitive outcome by SES.

**Table S14.** Binomial logistic regression analysis of the odds of a punitive outcome by HC.

**Table S15.** Binomial logistic regression analysis of the odds of a punitive outcome by HTT.

**Table S16.** Binomial Logistic Regression analysis of the odds of a punitive outcome by Total SVI for each Agency.

**Table S17.** Relevant Abbreviations.

| **ANOVA** | Analysis of Variance |
| --- | --- |
| **ATSDR** | Agency for Toxic Substances and Disease Registry |
| **CDC** | Centers for Disease Control and Prevention |
| **CI** | Confidence Interval |
| **DUA** | Data Use Agreement |
| **GIS** | Geographic Information Systems |
| **HC** | Household Characteristics Social Vulnerability Index Domain |
| **HTT** | Housing Type & Transportation Social Vulnerability Index Domain |
| **IQR** | Interquartile Range |
| **LFO** | Legal Financial Obligation |
| **M** | Mean |
| **OR** | Odds Ratio |
| **PNPOR** | Punitive/Non-Punitive Outcome Ratio |
| **Q** | Quartile |
| **RE** | Racial & Ethnic Minority Status Social Vulnerability Index Domain |
| **RQ** | Research Question |
| **SVI** | Social Vulnerability Index |
| **SES** | Socioeconomic Status Social Vulnerability Index Domain |
| **SD** | Standard Deviation |

*Note:* Abbreviations are listed in alphabetical order.
